# Hepatitis C Virus Activates a Neuregulin-Driven Circuit to Modify Surface Expression of Growth Factor Receptors of the ErbB Family

**DOI:** 10.1371/journal.pone.0148711

**Published:** 2016-02-17

**Authors:** Sabine Stindt, Patricia Cebula, Ute Albrecht, Verena Keitel, Jan Schulte am Esch, Wolfram T. Knoefel, Ralf Bartenschlager, Dieter Häussinger, Johannes G. Bode

**Affiliations:** 1 Department of Gastroenterology, Hepatology and Infectious Diseases, Medical Faculty, University Hospital, Heinrich Heine University of Düsseldorf, Moorenstrasse 5, 40225 Düsseldorf, Germany; 2 Department of General, Visceral, and Pediatric Surgery, Medical Faculty, University Hospital, Heinrich Heine University of Düsseldorf, Moorenstrasse 5, 40225 Düsseldorf, Germany; 3 Department for Infectious Diseases, Molecular Virology, Heidelberg University, Heidelberg, Germany; 4 Division for Virus-Associated Carcinogenesis, German Cancer Research Center (DKFZ), Heidelberg, Germany; University of Central Florida, UNITED STATES

## Abstract

Recently, the epidermal growth factor (EGF) receptor (EGFR), a member of the ErbB receptor family, and its down-stream signalling have been identified as co-factors for HCV entry and replication. Since EGFR also functions as a heterodimer with other ErbB receptor family members, the subject of the present study was to investigate a possible viral interference with these cellular components. By using genotype 1b replicon cells as well as an infection-based system we found that while transcript and protein levels of EGFR and ErbB2 were up-regulated or unaffected, respectively, HCV induced a substantial reduction of ErbB3 and ErbB4 expression. Down-regulation of ErbB3 expression by HCV involves specificity protein (Sp)1-mediated induction of Neuregulin (NRG)1 expression as well as activation of Akt. Consistently, at transcript level disruption of ErbB3 expression by HCV can be prevented by knockdown of NRG1 or Sp1 expression, whereas reconstitution of ErbB3 protein levels requires inhibition of HCV-induced NRG1 expression and of Akt activity. Interestingly, the NRG1-mediated suppression of ErbB3 expression by HCV results in an enhanced expression of EGFR and ErbB2 on the cell surface, which can be mimicked by siRNA-mediated knockdown of ErbB3 expression. These data delineate a novel mechanism enabling HCV to sway the composition of the ErbB family members on the surface of its host cell by an NRG1-driven circuit and unravels a yet unknown cross-regulation between ErbB3 and the two other family members ErbB2 and EGFR. The shift of the receptor surface expression of the ErbB family towards enhanced expression of ErbB2 and EGFR triggered by HCV was found to promote viral RNA replication and infectivity. This suggests that HCV rearranges expression of ErbB family members to adapt the cellular environment to its requirements.

## Introduction

The hepatitis C virus (HCV) still is one of the leading causes for chronic liver diseases worldwide. HCV broadly interferes with inter- and intracellular signaling pathways of the host involved in regulation of antiviral immunity and inflammatory response as well as in regulation of endocytosis, cell growth, apoptotic cell death and differentiation [1, 2]. Several signaling molecules of the host cell have been identified to be critical interaction partners for HCV proteins in order to subvert host antiviral effector mechanisms and to enable viral life cycle. Among others, this includes direct interaction of virus-encoded proteins with cellular signaling intermediates of the host or cleavage of key components of host cell signal transduction by the viral protease NS3/4A [1, 2]. A recently identified cellular substrate of NS3/4A is the ubiquitously expressed T-cell protein tyrosine phosphatase (TC-PTP) [3] and NS3/4A-mediated cleavage of TC-PTP induces a shift of the intrahepatic immune response towards a Th2-dominated immunity [4]. Moreover, emphasizing the *in vivo* relevance of this observation, NS3/4A protein levels and viral load inversely correlated with TC-PTP protein levels in individuals chronically infected with HCV [5]. Apart from this TC-PTP has been identified as an important endogenous negative regulator of the EGF Receptor (EGFR) [6, 7]. Consistently, NS3/4A-dependent cleavage of TC-PTP results in a sensitization of EGFR and an enhancement of ligand-induced activation of EGFR and EGFR-transmitted intra-cellular signal-transduction including enhanced activation of Akt and Phospholipase C (PLC)γ [3]. Down-regulation of TC-PTP expression levels by HCV not only results in an increased ligand-induced activation of Akt but also in a ligand- and EGFR-independent up-regulation of Akt activity, supporting viral replication [3]. It is likely that this NS3/4A-mediated sensitization of EGFR and EGFR signaling as well as the ligand-independent activation of Akt are somehow interlinked with the observation that EGFR is activated by HCV via cluster of differentiation (CD)81 binding and acts as a cofactor for HCV internalization and entry by promoting CD81-Claudin-1 complex formation [8, 9]. The fact that EGFR and EGFR-induced signaling are not only important for viral binding and internalization of HCV but also for other viruses and intra-cellular bacteria including influenza A virus [10] and *Chlamydia pneumonia* [11] suggests a more general role of EGFR for pathogen-host interaction and entry.

EGFR belongs to the ErbB family of receptor tyrosine kinases, consisting of four type 1 tyrosine kinase transmembrane glycoproteins that are structurally homologous and share highly conserved sequences. Apart from EGFR, also termed as ErbB1, or HER1, the ErbB-family includes ErbB2 (also known as HER2), ErbB3 (HER3) and ErbB4 (HER4), named after their ligands, Heregulins, also known as Neuregulins or NRGs [12]. In contrast to the other three members, the ErbB2 receptor is considered an orphan receptor since no known ligand binds to its extracellular domain. However, its tyrosine kinase domain is catalytically active, making it a co-receptor that can heterodimerize with the other ErbB family members to initiate signal transduction [13]. ErbB signal transduction is elicited upon ligand binding to the extracellular domain of the respective receptor molecule, which triggers hetero- or homodimerization amongst family members. This results in activation of the cytoplasmic tyrosine kinase domain and subsequent trans-phosphorylation of tyrosine residues within the cytoplasmic C-terminus of the receptor molecules. The phosphorylated tyrosine residues in turn represent recruitment sites for signaling intermediates leading to the activation of a complex signaling network, including the activation of extracellular signal-regulated kinase 1/2 (ERK1/2) and phosphoinositide 3-kinase (PI3K)/protein kinase B (AKT) pathways. The only exception within the ErbB receptor family is ErbB3 which lacks a functional tyrosine kinase domain [14]. Given that ErbB2 and ErbB3 lack part of the components required for signal transduction upon ligand binding, their main role is to act as co-receptors and heterodimerize with other ErbBs to elicit a cellular response from external stimuli. Being major regulators of cell division, cell death, differentiation and migration the ErbB receptor family is involved in the regulation of a variety of different physiological and pathophysiological conditions including organ development, growth, inflammation, wound-healing and regeneration [14]. Furthermore, up-regulation and aberrant signaling of ErbB receptor family members is considered to play a critical role in the pathogenesis of cancer [15].

While the role of EGFR for viral entry has been comparably well studied [8, 9] it is unclear in how far HCV interferes with the other ErbB family members. In the present study we analysed the impact of HCV on the expression of the other receptor family members and in particular on ErbB3 and the functional consequences of their regulation by HCV. Evidence is provided that HCV down-regulates expression of ErbB3 on both transcript and protein level and show that this is in part due to HCV-induced up-regulated expression of the ErbB3 receptor ligand Neuregulin (NRG)1. Apart from this HCV-mediated down-regulation of ErbB3 at the protein level also involves activation of Akt-dependent signaling. Interestingly, ligand-induced down-regulation of ErbB3 surface expression is accompanied by an increase in EGFR and ErbB2 surface expression, suggesting a paracrine mechanism in which NRG1 shedded by an HCV-infected cell renders surrounding cells more susceptible to HCV.

## Materials and Methods

### Materials

Antibodies: ErbB3 (1B2)(#4754), ErbB3 pTyr1289 (#4791), ErbB2 (#2165) and ErbB4 (#4795) were purchased from Cell Signaling (Danvers, USA). β-Aktin (#ab3280) and HCV NS3 (#ab18671) were obtained from Abcam (Cambridge, UK). GAPDH (#H86903M) was purchased from Biodesign (Memphis, USA). EGFR (#sc-03) was purchased from Santa Cruz (Santa Cruz, USA), HCV NS5A (Genotype 2a) for Western Blots was kindly provided by Ralf Bartenschlager. HRP-coupled Rabbit Anti-Mouse (#P0260) and Goat Anti-Rabbit (#P0448) antibodies were purchased from Dako (Agilent Technologies, Glostrup, Denmark). The anti Phycoerythrin-coupled EGFR antibody (#FAB10951P), the Allophycocyanin-coupled ErbB3/HER3 antibody (#FAB3481A) as well as the corresponding controls (Rat IgG2A Isotype Control Phycoerythrin Conjugated: #IC006P, Mouse IgG1 Isotype Control APC Conjugated: #IC002A) used for FACS were obtained from R&D Systems (Minneapolis, USA). The FITC-coupled ErbB2/Her2 antibody (#BMS120FI) and the corresponding control (FITC Conjugated Rat IgG2a Isotype Control: #11-4321-81) were purchased from eBioscience (San Diego, USA). MRP2 (#MA1-26536) was purchased from Thermo Fisher Scientific (Waltham, USA), Na^+^/K^+^ ATPase (#A276) from Sigma Aldrich (St. Louis, USA). HCV NS5A (Genotype 2a) (#HCM-131-5) for immunofluorescence staining was obtained from Austral Biologicals (San Ramon, CA). Human NRG1-β1/HRG1-β1 EGF Domain Antibody (#AF-396-NA) and Normal Goat IgG control (#AB-108-C) were obtained from R&D Systems (Minneapolis, USA). Inhibitors: Mithramycin A and 2-C’-Methylcytidin were purchased from Sigma Aldrich (Munich, Germany) and Triciribine was purchased from Merck Millipore (Darmstadt, Germany). Stimulants: NRG1-β1/HRG1-β1 EGF Domain was obtained from BioRad (Hercules, USA).

Cell culture media and fetal bovie serum were from Biochrom (Berlin, Germany) and from Perbio (Bonn, Germany), respectively. Glutamine, Penicillin, Streptomycin and NEAA was purchased from Invitrogen (Karlsruhe, Germany).

### Cell Culture

The human hepatoma cells Huh7 [16] as well as the Huh9-13 cell line harbouring the HCV subgenomic replicon genotype 1b [17] and the Huh21-5 cell line harbouring the full length replicon of HCV genotype 1b [18] were cultivated in Dulbecco’s modified Eagle’s medium/nutrient mix F-12 supplemented with 10% (vol/vol) heat-inactivated fetal bovine serum at 37°C in a humidified atmosphere with 5% CO_2_. The human hepatoma cell line Huh7.5 [19] was cultivated in Dulbecco’s MEM 4.5g/l Glucose supplemented with 9% (vol/vol) heat-inactivated fetal bovine serum, 2mM Glutamin, 100U/ml Penicillin, 100μg/ml Streptomycin, 10μl/ml non-essential aminoacids (MEM nonessential amino acids solution—Gibco, Thermo Fisher Scientific, Waltham, USA). Medium was changed 16 hours before experiments were performed. The use of these cell lines is covered by a material transfer agreement with Apath, L.L.C. (New York, USA).

### RNA isolation and Real-Time PCR

Total cellular RNA was isolated using the RNeasy Miniprep Kit (Qiagen, Hilden, Germany) according to manufacturer’s instructions. 1μg of total RNA was reverse transcribed with Quantitect Reverse Transcription Kit (Qiagen, Hilden, Germany) using oligo(dT), which included DNase I digestion. cDNA was diluted 1/5, and 1.2μl of the diluted cDNA was added as template to a final volume of 25μl including 1x SYBR Green PCR master mix (Applied Biosystems, Germany). The primers used are listed as a table provided in the supporting information (Table A in S1 File). No template and no reverse-transcriptase controls were used to control specificity of RT-PCR. Semi-quantitative PCR results were obtained using the ΔΔCT method. As control gene SDHA was used. Threshold values were normalized to SDHA. Data from at least three or more independent experiments are presented as means plus standard error of means (SEM).

### Transfection Procedure for Small Interfering RNA

Huh cells were transiently transfected using NRG1-, Sp1- or ErbB3-specific small interfering RNA (siRNA) from Thermo Scientific Dharmacon (Lafayette, CO) according to the manufacturer’s instructions. Briefly 5μl Dharmafect 4 was added to 195μl Optimem (Thermo Fisher Scientific, Walthan, USA) and in parallel 180μl Optimem was mixed with 20μl of a 5μM siRNA solution. After 5 minutes Dharmafect and siRNA Dilution were combined and after additional 20 minutes 1600μl of the respective culture medium was added. Cells were cultured in six well plates with 2 ml transfection medium per well. After six hours, transfection medium was exchanged against antibiotic free culture medium and culture was continued for additional maximum 72 hours. Medium was exchanged every 24 hours.

### Transfection Procedure for pCMV-Sp1 plasmid

Huh7 cells were transfected 24 hours after seeding into 6 well plates. For every well 2.5μg plasmid DNA and 50μl serum-free OptiMEM were mixed as well as 4μl Lipofectamin^**®**^ 2000 and 246μl serum-free OptiMEM. Both preparations were mixed and incubated for 30 minutes at room temperature. Afterwards, 1.7ml antibiotic-free medium was added and adherent cells were transfected by adding the transfection mix and incubating cells at 37°C for 48 hours. Medium was changed after 3 hours and 24 hours.

### Preparation of total cell lysates, Immunoblotting and Immunodetection

For the analysis of protein from total cell lysates, cells were solubilized in Triton lysis buffer (1% Triton, 20mM Tris/HCl, pH 7.4, 136mM NaCl, 2mM EDTA, 50mM β-glycerophosphate, 20mM sodium-pyrophosphate, 1mM Na_3_VO_4_, 4mM benzamidine, 0.2mM Pefabloc, 5μg/ml aprotinin, 5μg/ml leupeptin, 10% glycerol and 0.2% SDS) on ice. Lysates were vortexed and kept on ice for 10 minutes. Lysates were centrifuged for 15 minutes at 14.000rpm and 4°C. Supernatant was transferred and used for determination of protein concentration. 30μg of protein were subjected to SDS gel electrophoresis (10% PAA). The electrophoretically separated proteins were transferred onto polyvinylidene difluoride (PVDF) membranes by semidry Western blotting. Nonspecific binding was blocked with 5% bovine serum albumin (BSA) in TBS-T (20mM Tris/HCl, pH 7.4, 137mM NaCl, and 0.1% Tween) for 60 minutes. The blots were incubated overnight at 4°C with primary antibodies diluted in TBS-T. After extensive washing with TBS-T, blots were incubated with goat anti-rabbit IgG or rabbit anti-mouse IgG secondary antibodies conjugated to horseradish peroxidase and diluted in TBS-T for 1 hour at room temperature. After further rinsing in TBS-T, the immunoblots were developed with the enhanced chemiluminescence system (ECL) following the manufacturer’s instructions (PerkinElmer, MA, USA).

### Preparation and cultivation of Primary Human Hepatocytes

Human hepatocytes were isolated from liver samples (15 cm^3^) of tumor free liver tissue from patients undergoing partial hepatectomy for removal of liver metastasis after informed consent and in accordance with the guidelines of the Ethics Committee of the University of Düsseldorf, Germany, and the Declaration of Helsinki. A respective application has been provided to the Ethics Committee of the University Düsseldorf and has been approved (study number: 2852). The preparation and cultivation of human hepatocytes has been described elsewhere [20].

### Infection of Huh7.5 Cells with the HCV strain JC1 (HCVcc)

Huh7.5 cells were infected with HCV strain JC1 [21, 22] 24 hours after seeding with a multiplicity of infection (MOI) of 1. Cells were used for experiments 48 or 72 hours after infection. The percentage of infected cells was routinely analysed by assessment of NS5A expression using immunofluorescence (Fig B in S1 File). Infected cells were fixed in ice-cold Methanol and blocked in 5%FCS/PBS. Cells were detected by using a JFH1 NS5A-specific rabbit polyclonal antibody (dilution 1:200) and a Alexa Fluor 488-conjugated goat anti-rabbit antibody (Invitrogen, Karlsruhe, Germany) as a secondary antibody (dilution 1:500). Cell nuclei were visualized using Hoechst dye, cell membranes using the Na^+^/K^+^ ATPase. The percentage of infected cells was at 90% on average for acute and 60% on average for chronic infection.

### Differentation of Huh7.5 Cells

Differentiation and growth arrest of Huh7.5 was achieved using medium supplemented with 1% DMSO (vol/vol) as outlined previously [23]. Briefly, Huh7.5 cells were grown in 12-well plates. After 24 hours medium was changed to Huh7.5 medium supplemented with 2% DMSO (vol/vol). Medium was changed every 48 hours for 2 weeks. To check differentiation, cells were fixed in ice-cold methanol, and MRP2 (dilution 1:30) and the Na^+^/K^+^ ATPase (dilution 1:100) were stained as apical (canalicular) and basolateral (sinusoidal) marker proteins, respectively. Cell nuclei were visualized usind Hoecht dye (Fig C in S1 File).

### ELISA

NRG1 beta 1 Human ELISA Kit (#ab100614) was purchased from Abcam (Cambridge, UK). Cell culture supernatant was collected from 6 wells 24 hours after seeding. ELISA was performed according to the manufacturer’s instruction. In brief, coated ELISA plates were incubated with 100μl either un-diluted cell culture supernatants or un-diluted sera from patients and NRG1 beta 1 standard diluted according to the manufacturer’s instructions for 2.5 hours. Solution was discarted and wells were washed for 5 times with 200μl 1x Wash Solution. Afterwards, 100μl 1x Biotinylated NRG1 beta Detection Antibody was added and incubated for 1 hour. After an additional wash step, which was performed as outlined above, 100μl 1x HRP-Streptavidin solution was added and kept at room temperature for 45 minutes with gentle shaking. After one further wash step (see above) the wells were incubated with 100μl TMB One-Step Substrate Reagent for 30 minutes in the dark with gentle shaking. Reaction was stopped by addition of 50μl Stop Solution. Measurement followed immediately at 450nm.

### FACS analysis

Cells were seeded in 6 well plates for 48 hours or 96 hours in case of transfected cells. Cells were treated with 250μl Trypsin for 3min at 37°C and reaction was stopped by adding 1ml FACS buffer (483ml PBS w/o Ca^2+^/Mg^2+^, 10ml FCS, 2ml EDTA 0.5M). 500μl were transferred to FACS tubes containing 1ml FACS buffer. After centrifugation cells were fixed at room temperature for 15min and afterwards ErbB3-APC (Allophycocyanine)-, EGFR-PE (Phycoerythrine)- and ErbB2-FITC-antibodies or corresponding APC-, PE- and FITC-control antibodies were added according to the manufacturer’s instructions. Cells were put on ice for 30min. After two wash steps with FACS buffer cells were re-suspended in 250μl FACS buffer and measured using a FACSCanto II cytometer (BD Biosciences, Heidelberg, Germany). Analysis was performed with the FlowJo software (Version No 7.6.5).

### Immunofluorescence staining of infected cells

Cells on coverslips were washed twice with PBS before fixation with ice-cold Methanol for 30 seconds. After two wash steps with ice-cold PBS, fixed cells were blocked with 5% (v/v) FCS in PBS for 30 minutes and stained with a primary antibody in a dilution according to the manufacturor’s instructions for 1 hour at room temperature, followed by three wash steps with PBS for 10 minutes, and incubation with corresponding secondary antibodies for 1 hour at room temperature in the dark. After three wash steps for 10 minutes as above, traces of salt were removed by a short wash in demineralized water, and the coverslips were mounted using Dako Fluorescent Mounting Medium (Agilent Technologies, Glostrup, Denmark). Specimens were analysed with a LSM 510 Meta confocal laser scanning microscope (Zeiss, Jena, Germany).

### Image analysis

Confocal images were analysed using the cell counter plugin of the Fiji open-source platform for biological image analysis [24]. Three randomly chosen large fields of view were counted from three independent experiments.

### Statistical evaluation

The immunoblots were scanned and densitometric analysis was performed using ImageJ software from the national institutes of health, USA. Densitometric data were normalized against β- actin or GAPDH.

**Fig 1:**
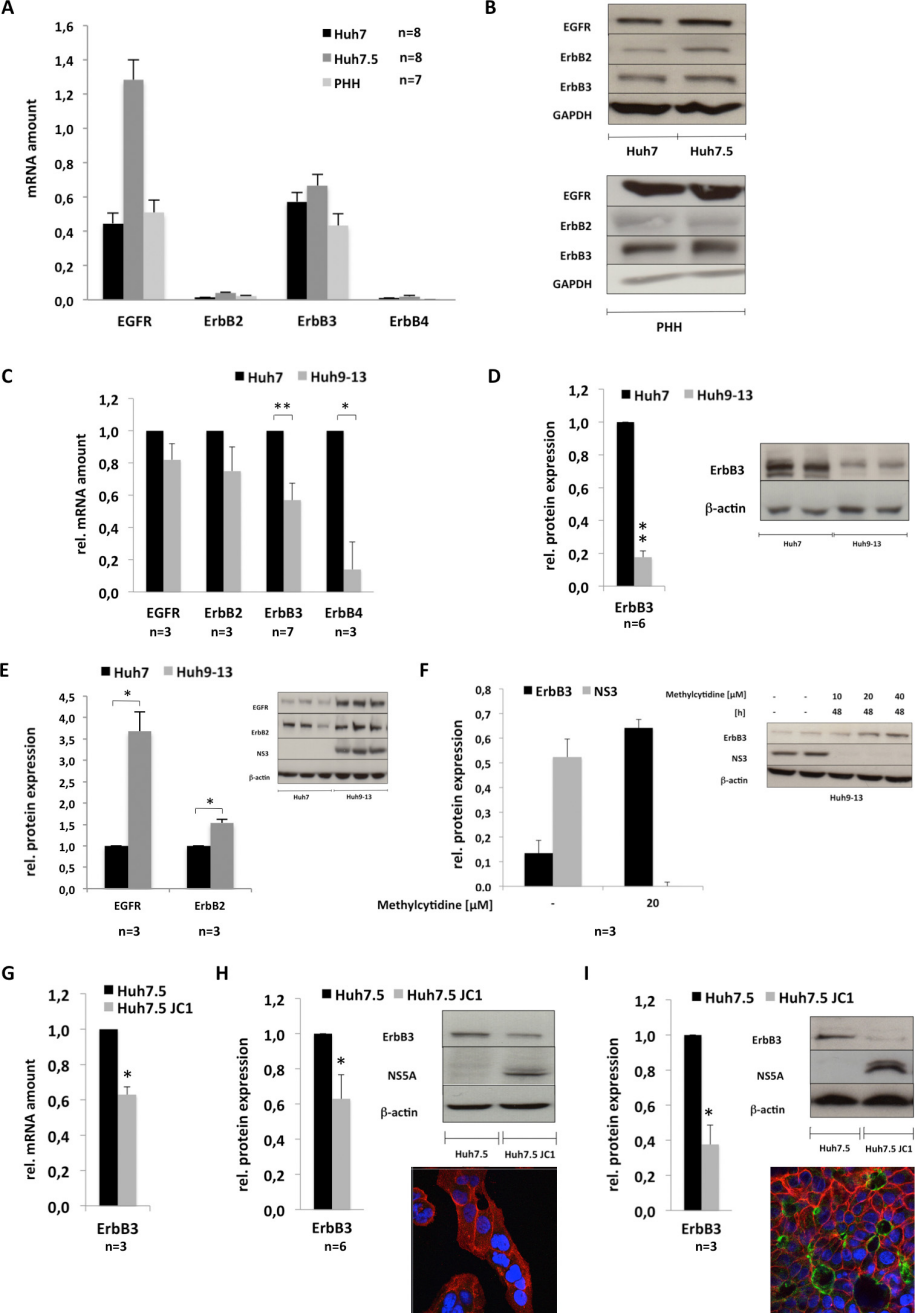
HCV suppresses expression of ErbB3 and modulates the expression of EGFR and ErbB2 to various extents. For (A) and (B) the hepatoma cell lines Huh7 and Huh7.5 were cultured for 48 hours while primary human hepatocytes (PHH) were isolated and cultured for 24 hours. Subsequently (A) total mRNA was analysed for transcript abundance of EGFR, ErbB2, ErbB3 and ErbB4 by rtPCR and for (B) total protein lysates were prepared and protein expression of EGFR, ErbB2 and ErbB3 was analysed by immunoblot using specific antibodies. β-actin or GAPDH levels were determined as loading controls. For (C) to (E) Huh cell lines harbouring the subgenomic replicon of HCV genotype 1b and the respective control cell line Huh7 cells were cultured for 48 hours and thereafter for (C) total RNA and for (D and E) total protein extracts were prepared and analysed for the abundance of EGFR, ErbB2, ErbB3 and ErbB4 transcripts (C) or for the protein levels of ErbB3 (D) as well as EGFR and ErbB2 (E). Additionally, NS3 expression was assessed for control of replication and β-actin levels for loading control. For (F) Huh9-13 cells were cultured for 24 hours and subsequently treated with 2-C’-Methylcytidine as indicated. After an additional 48 hours total protein extracts were prepared and analysed for ErbB3 and NS3 protein levels by immunoblot. (G and H) Huh7.5 cells were infected with 1 MOI of the HCVcc strain JC1 or left un-infected for control. Total RNA or protein lysates were prepared 48 hours after infection for quantification of the ErbB3 transcript (G) or 72 hours after infection for abundance of the respective protein by immunoblot using antibodies specifically recognizing ErbB3, NS5A or β-actin (H). For (I) Huh7.5 cells were differentiated by DMSO as summarized in the Material and Methods section and infected with the JC1 virus or left uninfected for control. Four weeks after infection total protein extracts were prepared and ErbB3 protein abundance was determined. To prove the differentiation status of the respective cell models used, Huh7.5 cells were either cultured for 5 days without DMSO treatment (H) or for 4 weeks in the presence of DMSO (I). After the respective culture period cells were fixed with ice cold methanol and analysed by immunofluorescence and confocal laser scanning imaging for the Na^+^/K^+^ ATPase as a basolateral marker (red) and for the presence of MRP2 which indicates polarization and formation of an apical compartment (green). Since MRP2 is only expressed in the apical membrane positive staining for MRP2 indicates cellular differentiation coinciding with polarization (compare immunofluorescence depicted in (H) with that in (I)). For (A), (C) and (G) semiquantitative rtPCR results were calculated using the ΔΔCT method and SDHA as control gene and for (A) data are presented as means + SEM of at least seven or more independent experiments. For (C) and (G) data are provided as fractions of the respective control cells which were set to one and are depicted as means + SEM of at least three independent experiments. The blots presented in (D) to (F), (H) and (I) were evaluated densitometrically and relative expression of ErbB3 (D,F,H and I), EGFR or ErbB2 (E) was normalized to β-actin and data are expressed as fractions of the normalized value of the respective control, which was set to one. Data are presented as means + SEM of at least three or more independent experiments. p ≤ 0.05 was considered to be significant.

Statistics were calculated using the SPSS Statistics software from IBM (Ehningen, Germany). The significance was calculated using the Mann-Whitney-U test and the Wilcoxon matched pairs test. Except for Fig 1A data are expressed as fractions of the normalized value of the respective control, which was set to one. Data are presented as means plus SEM (n≥3). P-values smaller than 0.05 were considered as significant. Data were marked with * for p≤0.05, ** for p≤0.01, or *** for p≤0.001.

## Results

### HCV down-regulates the expression of ErbB3 but not of EGFR or ErbB2 at the transcript and the protein level

While it is known that HCV influences the activity of the ErbB family member EGFR (Entrez Gene: 1956) and requires EGFR for entry and internalization [3, 8, 9], the impact of HCV on the other members of the ErbB receptor family is unknown. Therefore, the expression of the different ErbB receptor family members and the impact of the HCV subgenomic replicon on it was analysed at transcript and protein level in the hepatoma cell line Huh7 and compared to a Huh7-based cell line that is stably transfected with the sub-genomic HCV replicon genotype 1b (Huh9-13).

To determine baseline expression in the respective hepatoma cell lines Huh7 and Huh7.5 used for either the replicon system (Huh7) or the infection system (Huh7.5) expression of EGFR, ErbB2, ErbB3 and ErbB4 within both hepatoma cell lines was determined and compared to primary human hepatocytes (PHH). As shown in Fig 1A, at transcript level the expression profile of the different receptor family members in Huh7 and Huh7.5 cells was comparable to that observed in PHH with high levels of EGFR (Entrez Gene: 1956) and ErbB3 (Entrez Gene: 2065) and very low levels of the two family members ErbB2 (Entrez Gene: 2064) and 4 (Entrez Gene: 2066). Although the abundance of ErbB2 transcript was low in all cell lines, ErbB2 was, like EGFR and ErbB3, detectable at protein level by immunoblot (Fig 1B). In contrast, we were unable to detect ErbB4 in any of these cell lines by immunoblot using antibodies supposed to be specific for this receptor (data not shown). Due to this limitation, we decided to refrain from further analysis of the impact of HCV on the expression of ErbB4 and focused on the analysis of the molecular mechanisms and consequences of the down-regulation of ErbB3 expression by HCV which was observable at transcript (Fig 1C) and at protein level (Fig 1D) in cell lines bearing the sub-genomic HCV replicon (genotype 1b), when compared to the respective control cell line Huh7. Consistently, Fig 1C shows that in the presence of the subgenomic HCV replicon (Huh9-13), the expression of ErbB3 is significant down-regulated at transcript level (and also ErbB4), but not that of EGFR and ErbB2. In line with this observation, ErbB3 protein expression was also significantly reduced in cell lines harbouring a subgenomic replicon of HCV genotype 1b to approximately 20% of the expression level of respective control cells (Fig 1D). That this suppressive effect is indeed due to the presence of the HCV replicon is further substantiated by the fact that ErbB3 protein expression was rescued upon inhibition of replication using the NS5B inhibitor 2-C’-Methylcytidine (Fig 1F). The notion that HCV suppresses the expression of ErbB3 is further supported by the fact that ErbB3 expression was likewise reduced in Huh21-5 cells harbouring the full-length replicon (Fig A in S1 File) and in Huh7.5 cells infected with the HCVcc strain JC1 (Fig 1G and 1H) when expression was compared to Huh7 cells or un-infected Huh7.5 cells for control. Of note, down-regulation of ErbB3 expression in Huh7.5 cells infected with the HCVcc strain JC1 was even more pronounced when the recently described model of persistent infection was used (Fig 1I). This model is based on Huh7.5 cells, which upon long term treatment with DMSO differentiate into polarized cells expressing MRP2 and the Na^+^/K^+^ ATPase as apical and basolateral markers, respectively (Fig C in S1 File) and permit persistent infection in cell culture. These data suggest that suppression of ErbB3 expression occurs time-dependently and is maximal upon chronic infection. This is in line with the observation that in cell lines harbouring the HCV replicon ErbB3 suppression is as efficient as in the model of persistent infection (Fig 1C and 1I). In contrast to ErbB3, EGFR protein expression was strongly enhanced in the presence of the sub-genomic HCV replicon compared to Huh7 control cells while ErbB2 protein levels were slightly but significantly increased in cell lines harbouring the HCV replicon (Fig 1E).

### Down-regulation of ErbB3 expression by HCV involves HCV-induced up-regulation of NRG1 production and activation of Akt

**Fig 2:**
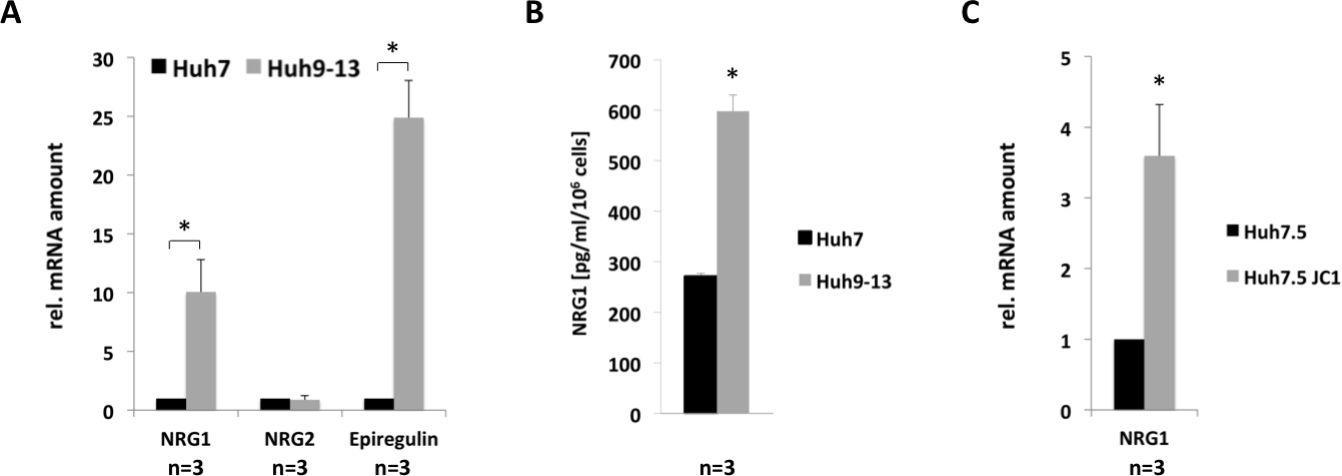
HCV induces the expression of the ErbB3 ligands NRG1 and Epiregulin. (A) Total RNA was isolated from Huh9-13 replicon cells or Huh7 control cells. Transcript levels of NRG1, NRG2 and Epiregulin were quantified by rtPCR as outlined in figure legend to Fig 1 and the Material and Methods section using primer pairs described in the supporting information (Table A in S1 File). (B) Supernatants from cell cultures of Huh9-13 cells comprising the subgenomic HCV replicon or Huh7 cells for control were analysed for production and release of soluble NRG1 using ELISA specifically recognizing human NRG1. (C) Huh7.5 cells were infected with the HCVcc strain JC1. Total RNA was prepared 72 hours after infection and NRG1 was determined by rtPCR. For (A) to (C) the results are expressed as fractions of the normalized value of the control which was set to 1; data are presented as mean + SEM of at least three independent experiments.

Recently, evidence was provided that Neuregulins can reduce the expression of ErbB3, which like Epiregulin (Entrez Gene: 2069), are ligands of ErbB3 [25]. To determine the influence of HCV on the expression of the different ErbB3 ligands Neuregulin (NRG)1 (Entrez Gene: 3084) and 2 (Entrez Gene: 9542) or Epiregulin, the expression of those ligands was assessed in cell lines harbouring the sub-genomic HCV replicon and Huh7 cells for control. As demonstrated in Fig 2 the presence of the HCV sub-genomic replicon resulted in an enhanced expression of both NRG1 and Epiregulin at transcript level, while NRG2 expression was not affected (Fig 2A). Accordingly, compared to the control cell line Huh7 the release of free NRG1 was enhanced more than two-fold in Huh9-13 cells when soluble NRG1 was determined in cell culture supernatants on protein level (Fig 2B). These data indicate that HCV infection results in an enhanced expression of NRG1. In line with this finding, infection of Huh7.5 cells with the HCVcc strain JC1 also led to enhanced expression of NRG1 as demonstrated in Fig 2C.

**Fig 3:**
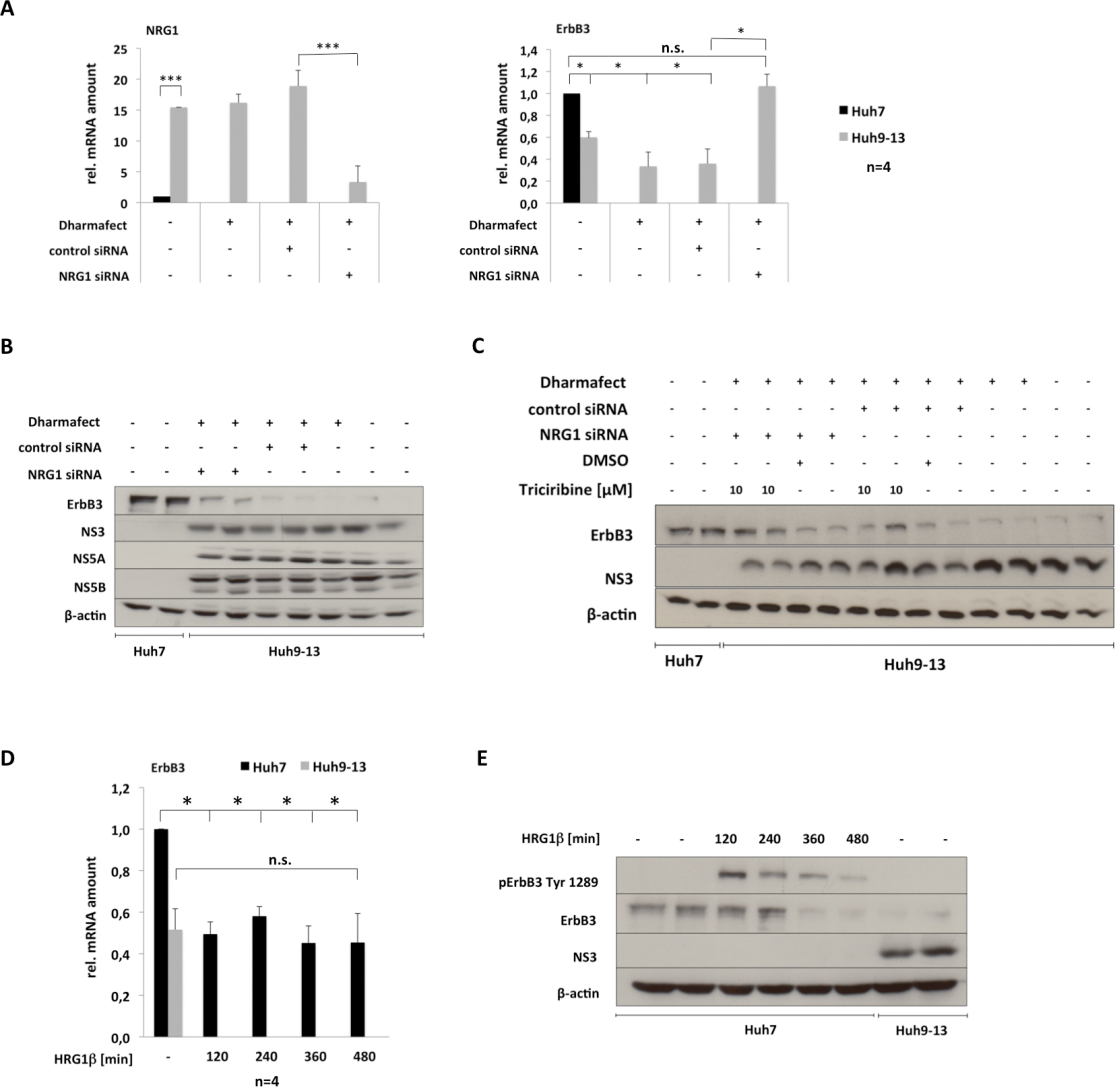
HCV NRG1-dependently down-regulates ErbB3 expression. (A) to (C) Huh 9–13 cells were transfected with control siRNA or NRG1-specific siRNA as outlined in the Materials and Methods section. 72 hours after transfection total RNA or protein was extracted and amounts of NRG1 and ErbB3 transcripts were quantified by rtPCR (A) as outlined in figure legend to Fig 1 and the Material and Methods section, whereas expression of the viral proteins NS5A and NS5B as well as of ErbB3 were determined by immunoblot (B). β-actin expression was assessed as loading control. (C) Knock-down of NRG1 expression was performed in Huh9-13 replicon cells using NRG1 specific siRNA or respective control siRNA as indicated and culture was continued for in total 72 hours after transfection. 10 μM Triciribine or DMSO that served as control were added 12 hours prior to the end of the total incubation period of 72 hours as depicted and total protein extracts were prepared to analyse the expression of ErbB3, NS3 and β-actin by immunoblot. (D) and (E) Huh7 cells were pre-treated with 25 ng/ml of the NRG1 isoform Heregulin 1β for the time periods indicated. Thereafter total RNA was isolated and the abundance of the ErbB3 transcript was assessed by rtPCR (D) while immunoblot was used to determine ErbB3 protein levels (E). For comparison, protein extracts from untreated Huh9-13 replicon cells were analysed in parallel. Results of at least 3 independent experiments are depicted. For (A) and (D) data were calculated as outlined in the legend to Fig 1 and data are presented as mean + SEM.

To investigate whether the HCV-mediated up-regulation of NRG1 is responsible for the down-regulation of ErbB3 expression observable in the presence of HCV, the expression of NRG1 was suppressed in cell lines harbouring the sub-genomic replicon of HCV using NRG1-specific siRNA. As shown in Fig 3A knockdown of NRG1 expression resulted in a complete reconstitution of ErbB3 transcript levels, when compared to respective controls, indicating that up-regulation of NRG1 expression plays a role for mediating the HCV-dependent reduction of ErbB3 transcript levels. However, while knockdown of NRG1 was sufficient to fully neutralize the inhibitory effect of HCV on the expression of the ErbB3 transcript, the expression of the protein was only partially restored (Fig 3B). This indicates that HCV engages additional pathways that are not sufficiently prevented by knockdown of NRG1 expression to hamper ErbB3 protein expression.

In this context it should be noted, that HCV also elicits a constitutive, ligand independent activation of Akt (Entrez Gene: 207) in its host cell [3]. Since Akt also mediates Nrdp1 (Entrez Gene: 10193)-dependent degradation of ErbB3 at protein level [25] and is known to be constitutively activated by HCV [3] the effect of the Akt-specific inhibitor Triciribine on HCV-mediated down-regulation of ErbB3 expression was analysed in the presence or absence of NRG1-specific siRNA. The results summarized in Fig 3C demonstrate that Akt inhibition alone is sufficient to partially restore ErbB3 expression at protein level, while complete rescue of ErbB3 proteins levels back to control levels requires combined inhibition of Akt activity and of NRG1 expression. These data strongly support the notion that the induction of the release of NRG1 by HCV is sufficient to explain the HCV-mediated down-regulation of ErbB3 expression at transcript level, whereas additional Akt-dependent signals, which are not exclusively triggered by NRG1, contribute to the suppressive effect of HCV on ErbB3 protein expression. However, Fig 3D and 3E show that treatment of Huh7 cells with 25 ng/ml of the NRG1 isoform Heregulin (HRG-)1β reduces ErbB3 expression at transcript and later on also at protein level, indicating that, at the concentrations used, NRG1 alone is likewise sufficient to mediate down-regulation of ErbB3 expression.

### The transcription factor Sp1 plays a potential role in up-regulation of NRG1 expression

**Fig 4:**
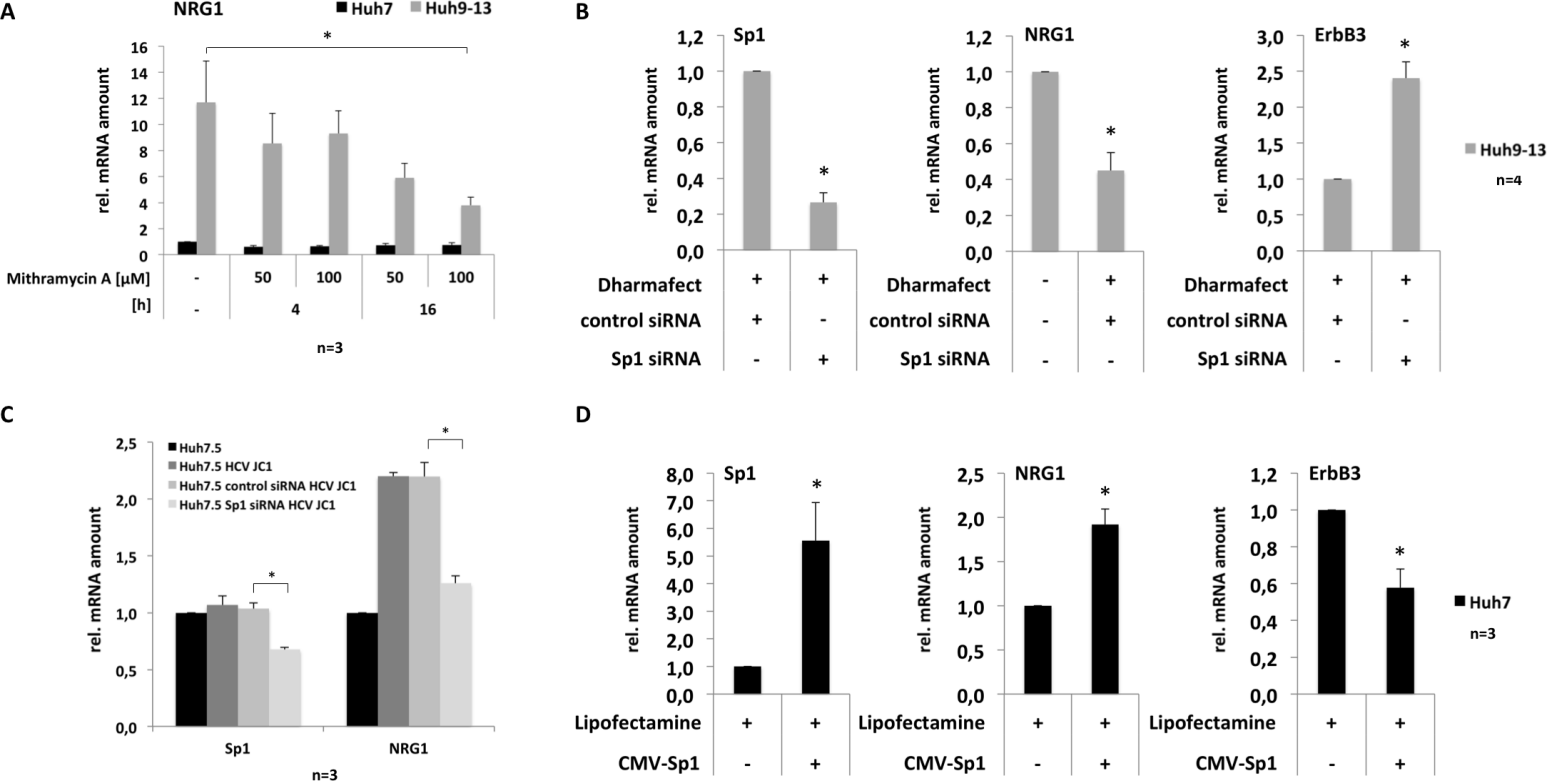
HCV mediated upregulation of NRG1 expression involves the transcription factor Sp1. (A) Huh7 or Huh9-13 cells were treated with Mithramycin A at the concentrations indicated or left untreated for control. Four and 16 hours after addition of Mithramycin A, total RNA was isolated and NRG1 mRNA expression was quantified by rtPCR. Values were normalized to those obtained from the control cell line Huh7. (B) Huh9-13 cells were transfected with a Sp1-specific siRNA. 72 hours later total RNA was isolated and abundance of Sp1, NRG1 and ErbB3 mRNA was quantified by rtPCR (left, middle and right subpanel, respectively) as outlined in the figure legend to Fig 1 and the Material and Methods section. Values were normalized to those obtained with control siRNA-transfected cells. (C) Huh7.5 cells were transfected with control siRNA or Sp1-specific siRNA as indicated and 48 hours later cells were infected with the HCV strain JC1 using a MOI of 1 TCID_50_/cell. After additional 48 hours total RNA extracts were prepared and amounts of Sp1 and NRG1 RNA was determined by rtPCR. (D) Huh7 cells were transfected with a Sp1-encoding plasmid and 48 hours later total RNA was extracted and amounts of Sp1-, NRG1- and ErbB3-specific transcripts were quantified by rtPCR. Semiquantitative PCR results were calculated as outlined in the legend to Fig 1 and data are presented as mean + SEM.

A potential mechanism by which HCV might up-regulate NRG1 expression is the activation of the transcription factor Sp1 (Entrez Gene: 6667), which is activated by HCV [26] and regulates *NRG1* gene expression via binding to respective GC-rich regions located within the 5´-regulatory region of the NRG1 gene [27]. To determine a potential involvement of Sp1 in the regulation of HCV-induced NRG1 production, both Huh7 and Huh9-13 replicon cells were treated with Mithramycin A. The compound binds to GC-rich DNA with high affinity, thereby displacing the Sp1 family proteins from binding sites blocking Sp1-dependent transcription [28, 29]. Fig 4A shows that treatment of Huh9-13 cells harbouring the HCV replicon with Mithramycin A results in down-regulation of NRG1 expression suggesting that Sp1 is indeed involved in HCV-mediated NRG1 up-regulation. In support of this, a siRNA knockdown of Sp1 in Huh9-13 replicon cells caused down-regulation of NRG1 expression, which coincided with up-regulation of ErbB3 expression (Fig 4B). Likewise, the Sp1 knockdown in Huh7.5 cells prior to infection with the HCVcc strain JC1 completely abolished virus-induced up-regulation of NRG1 expression (Fig 4C). Finally, over-expression of Sp1 in the hepatoma cell line Huh7 led to enhanced expression of NRG1 and resulted in a decreased expression of ErbB3 (Fig 4D). Taken together, these data strongly suggests that HCV Sp1-dependently mediates up-regulation of NRG1 expression and support the notion that enhanced expression of NRG1 and down-regulation of ErbB3 expression are functionally linked.

### Down-regulation of ErbB3 surface expression results in enhanced expression of EGFR and ErbB2 at the cell surface

**Fig 5:**
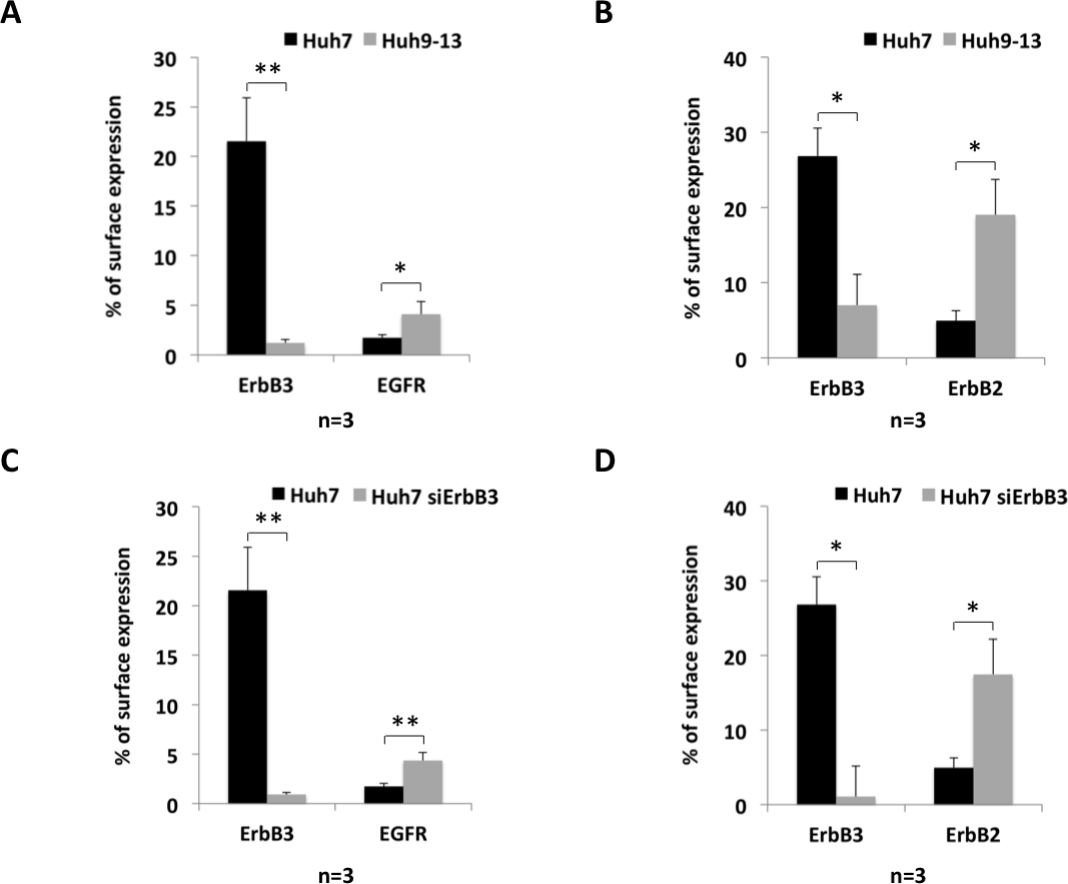
Reduction of ErbB3 expression on the cell surface is accompanied by increased cell surface expression of EGFR and ErbB2. (A) and (B) Huh9-13 replicon cells were analysed by FACS to determine surface expression of ErbB3 and EGFR (A) or of ErbB3 and ErbB2 (B). (C) and (D) Huh7 cells were transfected with ErbB3 specific siRNA or with control siRNA. 72 hours later cells were prepared for FACS analysis to determine the amounts of ErbB3 and EGFR (C) or of ErbB3 and ErbB2 (D) on the cell surface. Data were analysed using the FlowJo software against the corresponding isotype controls and analysis of EGFR (A and C) or ErbB2 (B and D) surface expression was gated to cells with low expression of ErbB3. Results correspond to the average of the receptors at the cell surface (in precent); error bars indicate SEM from at least 3 independent experiments.

To analyse the impact of HCV-mediated ErbB3 receptor reduction on the surface expression of the other ErbB family members, their surface-expression was determined by FACS. Analysis of ErbB4 (Entrez Gene: 2066) expression was elided, since ErbB4 was only detectable at the transcript level but not at the protein level. Interestingly, down-regulation of ErB3 receptor by the HCV replicon was accompanied by an increased cell surface expression of both EGFR and ErbB2 (Fig 5A and 5B). Likewise, ErbB3 siRNA knockdown in replicon-free Huh7 cells caused an enhanced cell surface expression of both EGFR and ErbB2 (Fig 5C and 5D), indicating that the up-regulated surface expression of both ErbB2 and EGFR is indeed the consequence of ErbB3 receptor reduction.

### Increased production of NRG1 and down-regulation of ErbB3 expression improves conditions for viral replication and/or infectivity

**Fig 6:**
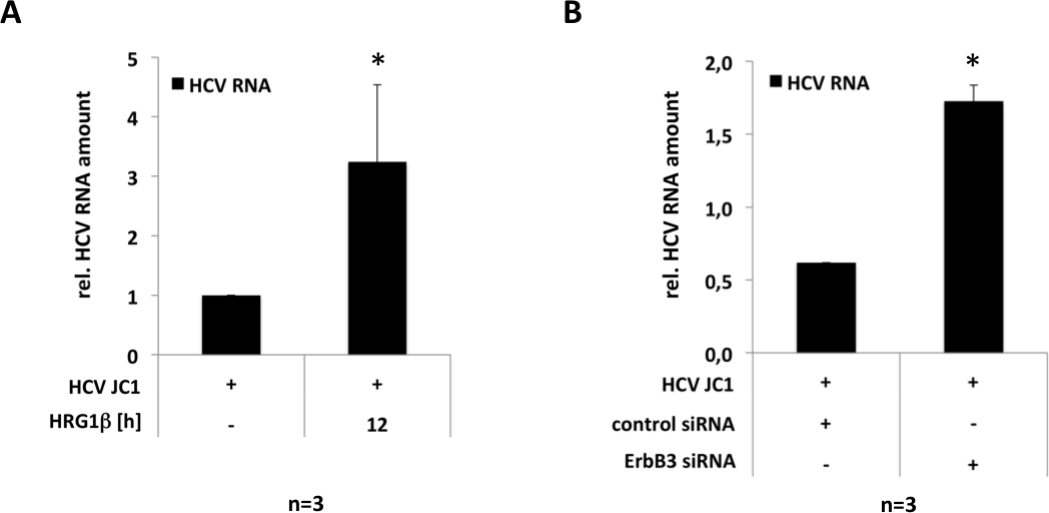
NRG1 and subsequent down-regulation of ErbB3 enhances replication of HCV. (A) Huh7.5 cells were pretreated with 25 ng/ml of the NRG1 homologue HRG-1β as indicated and subsequently infected with 1 MOI of the HCVcc strain JC1. Seventy-two hours after infection total RNA was prepared and abundance of the HCV genome was determined by rtPCR. (B) Huh7.5 cells were transfected with control siRNA or ErbB3-specific siRNA as described in Materials and Methods. Forty-eight hours after transfection cells were infected with the HCVcc strain JC1 using 1 MOI. After another 48 hours total RNA was extracted and analysed for the abundance of HCV RNA genomes and of succinate dehydrogenase complex subunit A (SDHA) by rtPCR. Semi-quantitative PCR results were calculated as outlined in the legend to Fig 1 from at least three independent experiments and are presented means + SEM.

**Fig 7:**
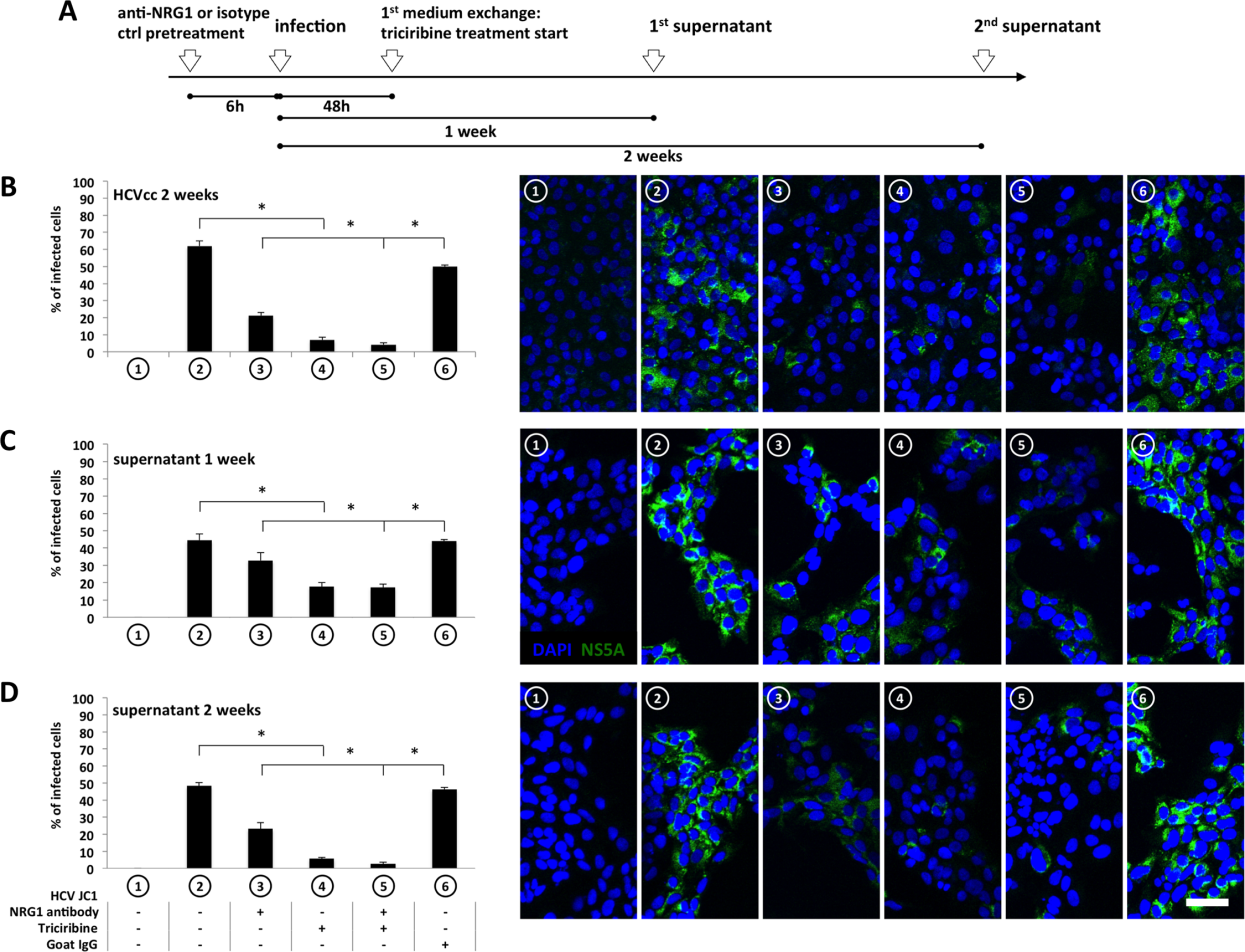
Inhibition of NRG1 and/or Akt activity results in an impaired establishment of infection of DMSO differentiated Huh7.5 cells and a reduced infectivity of conditioned supernatants. The experimental procedure and timeline is shematically summarized in (A). For (B) to (D) Huh7.5 cells were treated with 2% DMSO for two weeks prior to infection. Thereafter cells were infected with 2 MOI HCV JC1 for 48 hours according to the recently published protocols to establish persistent infection [23]. 6 hours prior to infection 2μg/ml of neutralizing NRG1 antibody or 2 μg/ml of the corresponding IgG isotype control were added to the cells as indicated. Medium was exchanged 48 hours post infection and afterwards every second day. With every exchange, 2μg/ml neutralizing NRG1 antibody or IgG control and/or 2μM Triciribine were replenished. For (B) cells were fixed two weeks after infection with ice-cold methanol and analysed for NS5A expression by immunofluorescence and laser scanning microscopy using antibodies specific for NS5A. To test for production of infectious virus the medium was changed for the last time either six (C) or thirteen (D) days after infection and incubation was continued for additional 24 hours. Thereafter supernatants were collected and transferred to fresh, untreated cells. After an incubation period of an additional 72 hours cells were fixed with ice-cold methanol and submitted to immunofluorescence analysis for NS5A expression as above. Cell nuclei were stained using Hoechst dye. Bar = 50μm. Confocal images in (B) to (D) are representative for the image sets taken to obtain quantitative data as shown in the respective column diagrams. For analysis cell nuclei and infected cells were counted using Fiji software and the percentage of infected cells is depicted as the mean and SEM from at least three independent experiments. p ≤ 0.05 was considered to be significantly different.

EGFR and activation of Akt-dependent growth factor signaling is important as co-factor for HCV infection and replication [8, 9]. The data presented herein suggest that HCV modulates surface expression of ErbB family members via enhanced production of NRG1, subsequently mediating down-regulation of ErbB3 expression followed by an increased surface expression of EGFR and ErbB2. To determine the impact of these changes elicited by enhanced production of NRG1 on viral replication and infectivity different experimental approaches were chosen. Hence, the impact of NRG1 treatment or down-regulation of ErbB3 expression on viral replication was determined. Moreover, using the experimental cell culture model of persistent infection, the effect of NRG1 neutralizing antibodies and / or pre-treatment with the Akt-inhibitor Triciribine on HCV infectivity was investigated. As depicted in Fig 6 pre-treatment of Huh7.5 cells with NRG1 (Fig 6A) as well as knockdown of ErbB3 expression using specific siRNA prior to infection with the HCVcc strain JC1 (Fig 6B) results in an increased abundance of viral RNA. This indicates that the cellular effects evoked by NRG1 and in particular the suppression of ErbB3 expression improve the conditions for viral replication. In support of the assumption that NRG1 and Akt-mediated effects may be beneficial for viral life cycle, treatment of DMSO-primed Huh7.5 cells with NRG1 antagonizing antibodies or with the Akt inhibitor Triciribine prior to infection with the HCVcc strain JC1 (Fig 7A) impedes HCV infection and reduces production of infectious particles. Consistently, as suggested from the quantification of NS5A positive cells two weeks after infection (Fig 7B), neutralization of NRG1 as well as inhibition of Akt or a combination of both substantially reduces the number of NS5A-expressing cells, when compared to respective IgG isotype or DMSO controls. Likewise, NS5A expression was strongly affected in cell cultures, which have been exposed to supernatants collected from HCV infected cell cultures in which NRG1-action was neutralized or activation of Akt was blocked for one or two weeks (Fig 7C and 7D) indicating a reduced infectivity of these supernatants when compared to respective control conditions.

## Discussion

Binding of HCV induces activation of the EGF receptor, which acts as a cofactor for viral entry and promotes formation of the CD81-Claudin-1 complex as well as viral replication [8, 9]. Once infected, the virus-encoded protease NS3/4A, among other cellular substrates [1], cleaves the protein tyrosine phosphatase TC-PTP, which is an important endogenous negative regulator of the EGF receptor [7], leading to a sensitization of EGFR [3]. This results in an enhanced ligand-induced EGF signaling in HCV infected cells and in a ligand-independent activation of Akt, which further promotes viral replication [3]. While the impact of HCV on EGFR has been adressed by several studies its influence on the expression and function of the other members of the ErbB receptor family is unknown.

Investigating the impact of HCV on the expression of the different members of the ErbB receptor family the data presented for the first time document that the presence of HCV mediates down-regulation of the ErbB receptor family members ErbB3 and ErbB4 (Fig 1). Since ErbB4 expression was only detectable at transcript but not at protein levels the work was focused on the mechanisms and consequences of down-regulation of ErbB3 expression in the presence of HCV. The data indicate that down-regulation of ErbB3 expression in HCV infected cells is due to a NRG1-mediated feedback loop triggered by HCV (Fig 3) resulting in a shift towards an increased surface expression of EGFR and ErbB2 (Fig 5). This effect of HCV on surface expression of EGFR and ErbB2 can be mimicked independent from HCV by siRNA-mediated knockdown of ErbB3 expression (Fig 5C and 5D). Hence, down-regulation of ErbB3 expression by HCV is indeed responsible for the observed changes with enhanced surface expression of EGFR and ErbB2 (Fig 5A and 5B). These data delineate a novel mechanism enabling HCV to sway the composition of the ErbB family members on its host cell by an NRG1-driven circuit. Furthermore, they for the first time suggest an interdependency of the surface expression of the different family members of the ErbB receptor family with an increased surface expression of EGFR and ErbB2 upon down-regulation of ErbB3 protein levels (Fig 5C and 5D). The enhancement of NRG1 production as well as the down-regulation of ErbB3 expression in turn appears to enhance viral replication and infectivity (Figs 6 and 7).

**Fig 8:**
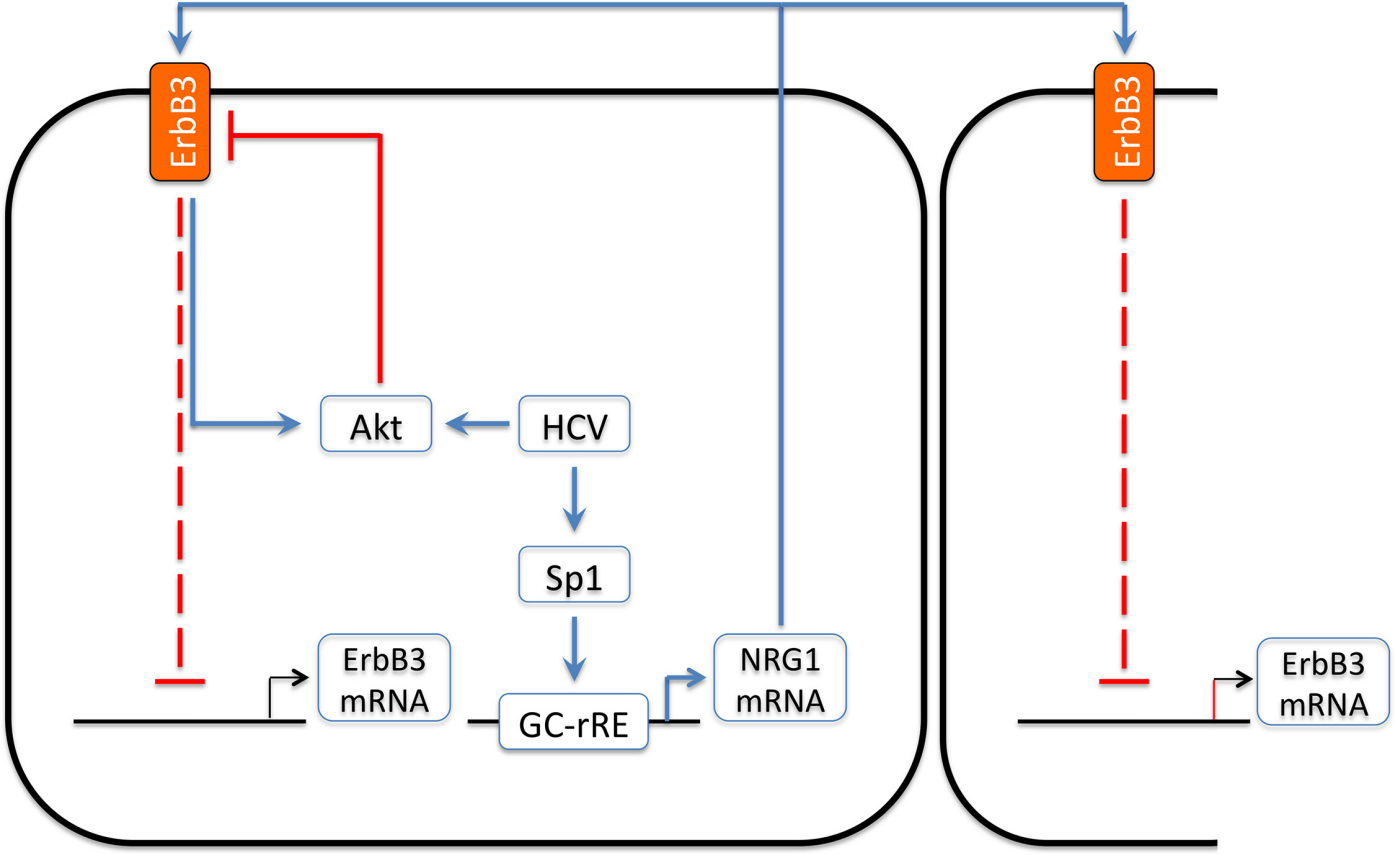
Schematic summary of the proposed mechanisms by which HCV mediates down-regulation of ErbB3 Expression. HCV up-regulates production of the ErbB3 ligand NRG1 (see also Fig 2) in its host cell by a mechanism that involves Sp1 (see also Fig 4). NRG1 in turn mediates down-regulation of ErbB3 transcript expression via an Akt-dependent pathway, which is also ligand-independently activated by HCV itself, and also of ErbB3-protein levels (see also Fig 3). Points which are not part of the scheme are that down-regulation of ErbB3 expression leads to an up-regulation of EGFR and ErbB2 expression by yet unknown mechanisms and promotes viral replication and infectivity.

The molecular mechanisms engaged in the regulation of NRG1 gene expression are incompletely understood. A recent characterization of the 5´ regulatory region of the NRG1 gene identified the prevalence of GC- and GT-box elements within a promoter region of the NRG1 gene that is highly conserved among mammals [27]. In particular the GC-box elements represent known binding sites for the zinc-finger domain-containing Sp-family of transcription factors [30], which are also activated by HCV as demonstrated previously for the Sp-family member Sp1 [26]. In this context the observation that treatment with Mithramycin A results in a substantial reduction of NRG1 transcript expression in cell lines that harbor the HCV sub-genomic replicon was interesting, since Mithramycin A binds with high affinity to GC-rich DNA and thereby displaces Sp-family proteins from respective binding sites [29]. These data suggest that activation of Sp-family members plays a role for HCV-mediated induction of NRG1 gene expression (Fig 8). This assumption is further supported by the fact that knockdown of Sp1 expression in cells harboring the sub-genomic replicon of HCV prevents enhanced expression of NRG1 (Fig 4B), while transient transfection of Huh7 cells with Sp1 results in an increased NRG1 expression (Fig 4D). In line with this, knockdown of Sp1 prior to infection prevented HCV-mediated up-regulation of NRG1 expression (Fig 4C) suggesting that HCV Sp1-dependently mediates increased production of NRG1. Thereby, suppression or induction of NRG1 expression as a result from the respective manipulations of the Sp1 expression levels coincided either with a rescue of ErbB3 expression (Fig 4B) or with a reduced expression of ErbB3 (Fig 4D), respectively, indicating that HCV-mediated Sp1-dependent control of NRG1 expression and regulation of ErbB3 expression are functionally linked (Fig 8).

As confirmed herein by the treatment of Huh7 cells with the NRG1 homologue Heregulin 1β (Fig 3D and 3E), NRG1 is able to reduce the expression of its own receptor Erb3 [25, 31]. These data further support the assumption that the enhancement of NRG1 expression by HCV is responsible for the reduction of ErbB3 expression observed in cell lines which either harbor the sub-genomic (Figs 1 and 3C) or full-length (Fig A in S1 File) HCV replicon of genotype 1 or are infected with the HCVcc strain JC1 (Fig 1G to 1I) derived from genotype 2. In line with this, knockdown of NRG1 using specific siRNA results in a complete recovery of ErbB3 at transcript levels (Fig 3A) in cell lines harbouring the sub-genomic replicon. However, apart from precluding NRG1 expression, full reconstitution of ErbB3 expression at protein levels in the presence of HCV additionally requires inhibition of Akt (Fig 3C), which is constitutively activated in the presence of HCV as indicated from previous reports [3, 32]. Hence one may conclude from these data that in addition to the enhanced NRG1 production, activation of Akt is required to attain maximal down-regulation of ErbB3 expression by HCV at both, transcript and protein levels (Fig 8). The effect of Akt may be due to the fact that Akt mediates activation of the E3 ubiquitin ligase Nrdp1, which is required for NRG1-induced ubiquitinylation and ubiquitin-dependent degradation of ErbB3 [25].

With respect to NRG1 it has to be noted that NRG1 is expressed as the membrane-bound precursor pro-NRG1, which has to be further processed to soluble NRG1. In this context the metalloprotease ADAM17 has been identified as the major sheddase, which processes membrane-bound pro-NRG1 into soluble NRG1 [33]. The circumstance that the release of soluble NRG1 requires metalloproteinase-dependent cleavage may also be responsible for the high difference of HCV-induced up-regualtion of NRG1 at transcript level, which is about 10 fold (Fig 2A) and that of the processed, soluble protein, which is only up-regulated by about two fold (Fig 2B).

Previously evidence has been provided that the ErbB family of growth factor receptors and in particular the EGF receptor [8, 9, 34] as well as molecules, that are involved in down-stream signaling of members of the ErbB receptor family, such as Akt, Ras and MKNK1 [3, 35–37] play a role for the viral life cycle of HCV. Thereby EGFR is not only CD81-dependently activated by HCV and acts as a cofactor for viral entry, but, upon successful infection, is also sensitized towards ligand-mediated activation through NS3/4A-dependent cleavage of the tyrosine phosphatase TC-PTP, one of the major negative regulators of EGFR signalling. The latter not only results in an increased sensitivity of EGFR towards its ligands, but also in a ligand-independent activation of Akt, which can be blocked by treatment with Triciribine. This Triciribine-sensitive activity of Akt has been demonstrated to play a role for maintenance of viral replication [3], an observation, which has been also confirmed by overexpression of a constitutive active mutant of Akt [37] and is also supported by the observation reported herein that pretreatment with Triciribine inhibits infection and production of infectious HCV particles (Fig 7).

Stimulation of Huh7.5 cells with the NRG1 homologue Heregulin 1β enhances the abundance of viral transcript (Fig 6) while treatment with an antibody that neutralizes NRG1 prior to infection with the HCVcc strain JC1 (Fig 7A) inhibits establishment of infection (Fig 7B) and production of infectious particels from cell culture as suggested from supernatant transfer experiments one or two weeks after infection (Fig 7C and 7D). This together with the fact that siRNA-mediated knockdown of ErbB3 expression, results in an enhanced abundance of viral RNA suggests that NRG1-dependent down-regulation of ErbB3 expression and viral life cycle are functionally linked. In this context, the observation reported here, that HCV sways surface expression of the different family members of the ErbB family by NRG1-dependent down-regulation of ErbB3 is of particular interest since the resulting up-regulation of EGFR and ErbB2 (Fig 5A and 5B) may be in advantage for the viral life cycle. Consequently, in the light of the observation that the ErbB family member EGFR promotes viral entry and that EGFR signalling enhances viral replication [3, 8, 9], one may speculate that in its host HCV triggers the release of NRG1 in order to promote virus production and to facilitate infection of neighbouring cells. Indeed, preliminary data suggest that serum levels of soluble NRG1 tend to be increased in sera of patients chronically infected with HCV when compared to healthy individuals or to patients which were cured from chronic HCV infection (Fig D in S1 File). However, these data are yet preliminary and require further substantiation. Likewise, the molecular mechanisms that interlink down-regulation of ErbB3 expression to the up-regulation of EGFR and ErbB2 surface expression and the mechanisms by which these events promote viral life cycle remain to be established.

## Supporting Information

S1 File S1 File comprising the supporting Information. Table A. Sequences for realtime PCR primer pairs. Fig A. Reduced expression of ErbB3 in Huh7 cells containing a selectable HCV genome. Fig B. Infection efficiency of Huh7.5 cells with the JC1 virus. Fig C. Differentiation of Huh7.5 cells. Fig D. NRG1 levels tend to be elevated in sera of HCV infected patients.
(PDF)

## Abbreviations

ErbB: avian erythroblastosis oncogene B
EGFR: epidermal growth factor receptor
HCV: Hepatitis C virus
Sp: specificity protein
NRG: Neuregulin
NS: non-structural protein
Huh: human hepatoma cell line
HRG: Heregulin
HCVcc: cell culture-derived HCV
