# Supplementary material for: Hepatitis C Virus Activates a Neuregulin-Driven Circuit to Modify Surface Expression of Growth Factor Receptors of the ErbB Family
Source: PLoS One. 2016 Feb 17;11(2):e0148711. doi: 10.1371/journal.pone.0148711 (PMC4757098; doi:10.1371/journal.pone.0148711)
Supplement: S1 File — Table A. Sequences for realtime PCR primer pairs. Fig A. Reduced expression of ErbB3 in Huh7 cells containing a selectable HCV genome. Fig B. Infection efficiency of Huh7.5 cells with the JC1 virus. Fig C. Differentiation of Huh7.5 cells. Fig D. NRG1 levels tend to be elevated in sera of HCV infected patients. (PDF) [file pone.0148711.s001.pdf]

S1 FILE (SUPPORTING INFORMATION)

Hepatitis C Virus activates a Neuregulin-driven circuit to modify surface expression of growth factor receptors of the ErbB family

Sabine Stindt[1], Patricia Cebula[1], Ute Albrecht[1], Verena Keitel[1], Jan Schulte am Esch[2], Wolfram T. Knoefel[2], Ralf Bartenschlager[3,4], Dieter Häussinger[1], Johannes G. Bode[1*]

[1]Department of Gastroenterology, Hepatology and Infectious Diseases, Medical Faculty, University Hospital, Heinrich Heine University of Düsseldorf, Moorenstrasse 5, 40225 Düsseldorf, Germany

[2]Department of General, Visceral, and Pediatric Surgery, Medical Faculty, University Hospital, Heinrich Heine University of Düsseldorf, Moorenstrasse 5, 40225 Düsseldorf, Germany

[3]Department for Infectious Diseases, Molecular Virology, Heidelberg University, Heidelberg, Germany

[4]Division for Virus-Associated Carcinogenesis, German Cancer Research Center (DKFZ), Heidelberg, Germany

* Corresponding author:

E-mail: johannes.bode@med.uni-duesseldorf.de (JGB)

S1 File: supporting information to the manuscript "Hepatitis C Virus activates a Neuregulin-driven circuit to modify surface expression of growth factor receptors of the ErbB family". This file comprises a table which provides the sequences for the primer pairs used for rtPCR **(Table A)**. Furthermore, data are provided, which demonstrate that expression of the ErbB receptor family member ErbB3 is reduced in Huh7 cells harboring a selectable full length genome of HCV when compared to control cells **(Figure A)**. Moreover, the efficiency of the infection of Huh7.5 cells with the JC1 Virus is shown by immunofluorescence **(Figure B)** and that DMSO induces cellular polarization of Huh7.5 cells as indicated by positive staining for the transporter protein MRP2 indicating the formation of an apical membrane **(Figure C)**. Finally this file provides data on NRG1 expression in sera of HCV infected patients compared to healthy controls or to patients were HCV infection has been successfully eradicated **(Figure D)**.

TABLE A

EGFR for<br>EGFR rev	5’ catccagtggcgggacatag 3’<br>5’ gggacagcttggatcacact 3’
ErbB2 for<br>ErbB2 rev	5’ tgccgttggggttcctcct 3’<br>5’ aggactggcagggagtcgca 3’
2ErbB3 for<br>2ErbB3 rev	5‘ gcgccagctccgcttgactc 3‘<br>5‘ tcggtccctcacgatgtccct 3‘
ErbB4 for<br>ErbB4 rev	5’ tgggcaacctggagataacc 3’<br>5’ ccagaggcaggtaacgaaact 3’
NRG1 for<br>NRG1 rev	5‘ tcaagtggttcaagaatgggaa 3‘<br>5‘ atcagccagtgatgctttgt 3‘
NRG2 for<br>NRG2 rev	5’ gcccttcctaccgttggttca 3’<br>5’ tcctccaccttcaccttgttg 3’
EREG for<br>EREG rev	5’ tgtagctctgacatgaatggct 3’<br>5’ aagtgttcacatcggacacca 3’
NS5A JFH-1 for<br>NS5A JFH-1 rev	5’ ccgttgctggttgtgctct 3’<br>5’ gttgctggagggcttctgat 3’
Sp1 for<br>Sp1 rev	5’ gcacctgcccctactgtaaa 3’<br>5’ cactttcccacagccttgga 3’
hSDHA for<br>hSDHA rev	5‘ agatgtggtgtctcggtcgat 3‘<br>5‘ cgtgatctttctcagggcca 3‘

S1 File Table A. Primer pairs used for realtime PCR.

Supplementary Figures

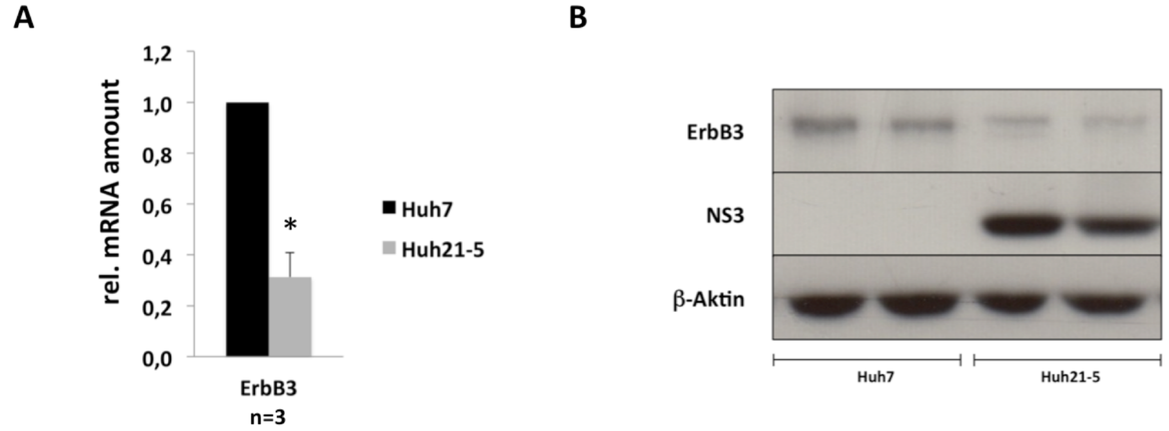

S1 File Fig A. Reduced expression of ErbB3 in Huh7 cells containing a selectable HCV genome. Hepatoma cells containing the genotype 1b-derived selectable HCV genome (Huh21-5 cells) and corresponding naive Huh7 cells were used. **A)** Total RNA extracts were prepared and subjected to real-time PCR to determine the abundance of the respective mRNA and succinate dehydrogenase complex subunit A (SDHA). Semiquantitative PCR results were calculated as outlined in Fig. 1 and are presented as the mean + SEM (n=3). **B)** Thirty µg of total protein lysates were seperated by SDS-PAGE using a 10% separation gel and subsequently analyzed by immunoblot using the respective antibodies. Western blots were treated with *ReBlot Plus Strong Stripping Solution* (Merck Millipore) between detection with different antibodies. Expression of β-actin was analyzed using a specific antibody and was used as loading control.

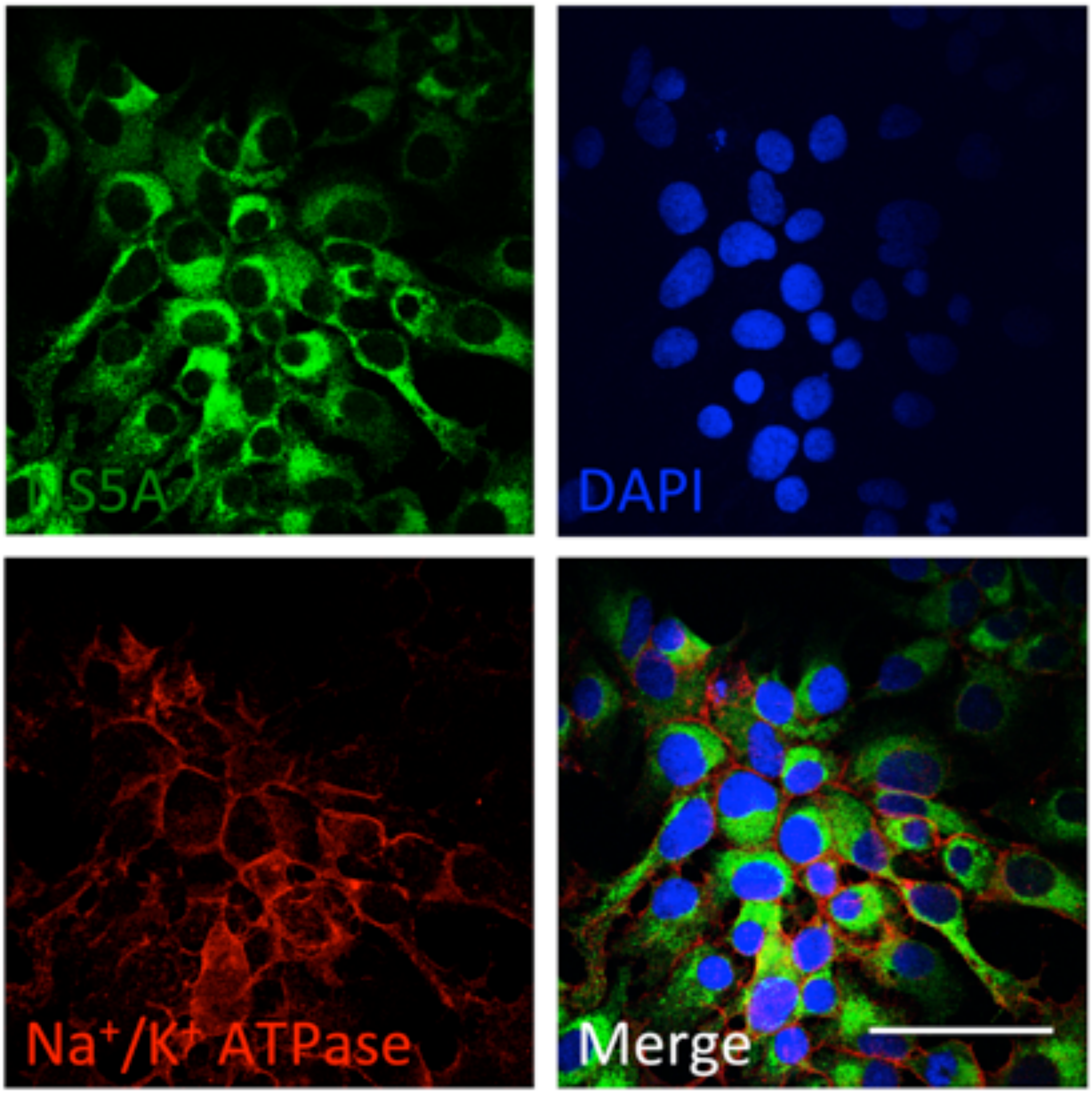

S1 File Fig B. Infection efficiency of Huh7.5 cells with the JC1 virus. Huh7.5 cells were infected with 1 MOI HCV JC1 for 72 hours and subsequently fixed in ice-cold methanol. NS5A was stained as viral marker protein as outlined in the material and methods section. The Na^+/K^+ ATPase was stained as basolateral (sinusoidal) marker protein, respectively. Cell nuclei were visualized using DAPI dye. Confocal laser scanning microscopy was performed using the confocal LSM 510 from Zeiss. Bar = 50µm

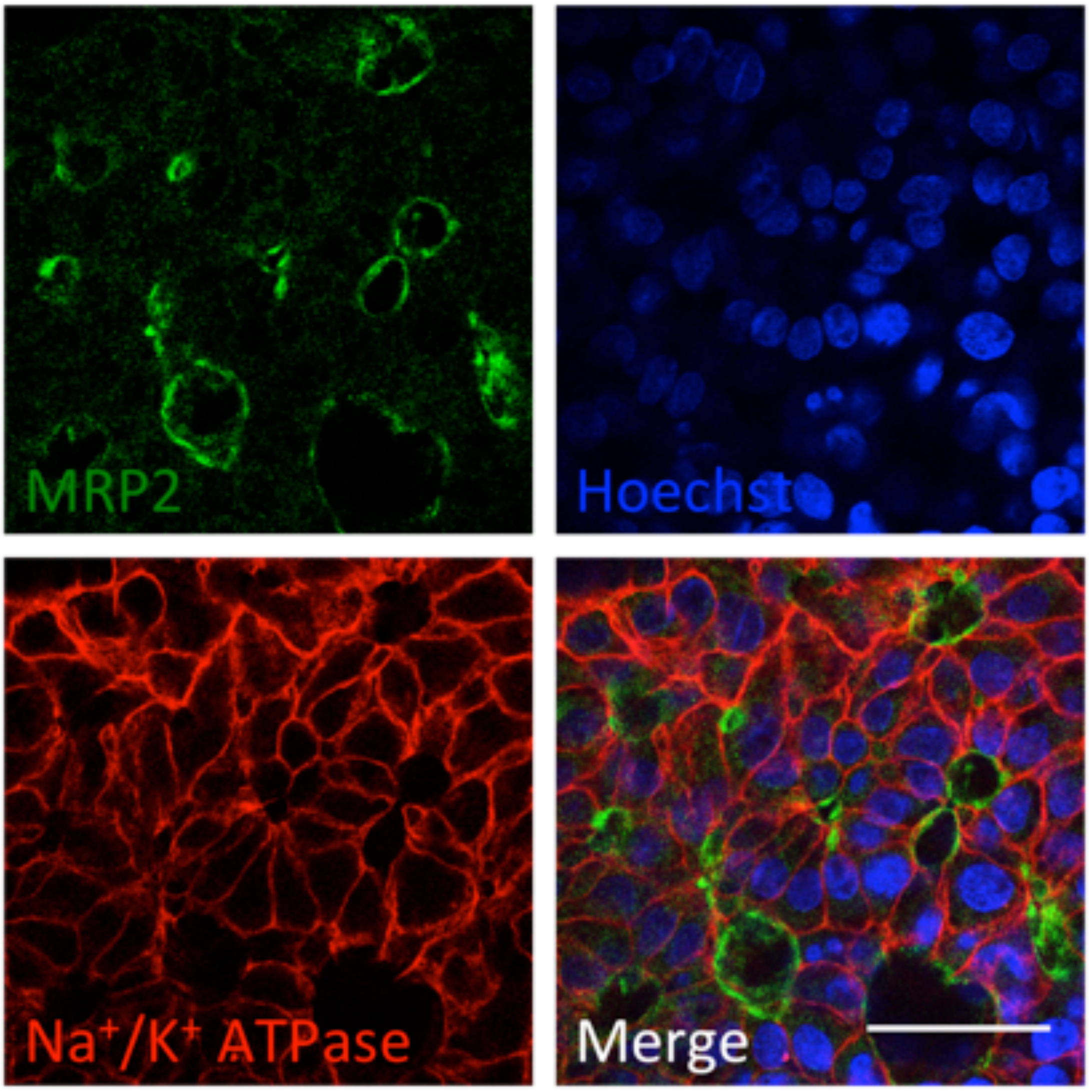

S1 File Fig C. Differentiation of Huh7.5 cells. Huh7.5 cells were treated with DMSO for four weeks which results in profound changes of their differentiation and polarization as suggested from the apical and basolateral marker proteins MRP2 and the Na^{+}/K^{+} ATPase. Subsequent to the respective differentiation period cells were fixed in ice-cold methanol, and MRP2 and the Na^{+}/K^{+} ATPase were stained as apical (canalicular) and basolateral (sinusoidal) marker proteins, respectively. Cell nuclei were visualized using Hoechst dye. Confocal laser scanning microscopy was performed using the confocal LSM 510 from Zeiss. Bar = 50µm

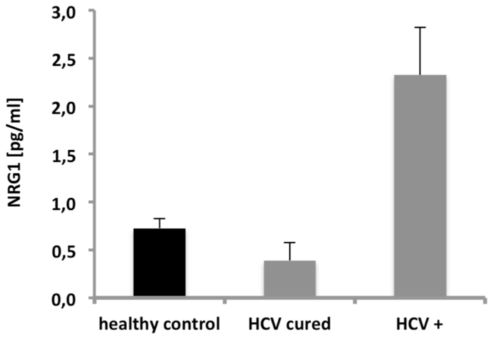

	control	HCV cured	HCV +
age	30±2	48 ± 6	59 ± 4
viral RNA	-	<12 U/ml	2.6 ± 1.3 x 10^6 U/ml
sex m/f	4/0	5/1	8/6

S1 File Fig D. NRG1 levels tend to be elevated in sera of HCV infected patients. Sera from healthy people (control), patients cured from HCV infection (viral load < 12 U/ml) and patients with chronic HCV infection (HCV +) were analyzed for the serum-concentrations of soluble NRG1 using ELISA as outlined in the Material and Methods section. Serum sampels were taken after informed consent and in accordance with the guidelines of the Ethics Committee of the University of Düsseldorf, Germany, and the Declaration of Helsinki. The table provides information on average age, sex distribution and viral load of the different groups.
